# Ultrasound‐Assisted Endoscopic Lesser Trochanter Resection for Ischiofemoral Impingement

**DOI:** 10.1002/atn2.70270

**Published:** 2026-11-11

**Authors:** Yuma Mitsuya, Kazuma Miyatake, Yohei Kusaba, Hirokazu Arakawa, Shinya Tsujiku, Yutaka Inaba

**Affiliations:** ^1^ Department of Orthopedic Surgery Yokohama City University Yokohama Kanagawa Japan

## Abstract

Ischiofemoral impingement is a rare cause of buttock pain resulting from narrowing of the space between the ischial tuberosity and the femoral lesser trochanter. Although most cases improve with conservative treatment, refractory cases may require surgical treatment. One such treatment option includes endoscopic resection of the lesser trochanter, which is an uncommon, extra‐articular, endoscopic surgery that carries several complication risks, such as injury of important neurovascular structures. By marking the sciatic nerve preoperatively and making a portal under ultrasound guidance, this surgery can be performed safely and easily.

## Graphical abstract

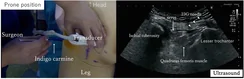

**VIDEO 1:** A detailed display of the surgical technique is shown in Video. Throughout the video, the patient is positioned prone, and the arthroscope is inserted through either the viewing portal or the medial portal. The portal used is indicated on the screen at each step. Video content can be viewed at https://doi.org/10.1002/atn2.70270.

Ischiofemoral impingement (IFI) is a rare cause of buttock pain because of narrowing of the ischiofemoral space (IFS) between the ischial tuberosity and the femoral lesser trochanter.^1^ IFI is difficult to diagnose because it is often confounded with lumbar spine disorders or intra‐articular hip pathologies.^2^ A local injection into the IFS can serve as a useful tool for the diagnostic treatment of IFI. Alternatively, surgical treatment is indicated for cases unresponsive to conservative treatment,^3^ and endoscopic resection of the lesser trochanter has been reported as an effective treatment option for decompressing IFS. Unlike standard intra‐articular arthroscopy, extra‐articular endoscopic procedures lack clear anatomic landmarks and require advanced skills to be performed safely and accurately.

In recent years, ultrasound‐assisted surgery—allowing real‐time visualization of nerves and vessels—has gained attention as a method to address this challenge. The use of ultrasound enables safe and minimally invasive maneuvers that are less dependent on the surgeon's experience.^4^, ^5^ In endoscopic lesser trochanter resection, ultrasound guidance can greatly enhance the safety and ease of the technique.

Furthermore, preoperative marking of the sciatic nerve with indigo carmine facilitates intraoperative nerve identification. This technical note describes ultrasound‐assisted endoscopic lesser trochanter resection for IFI, including the method used for preoperative sciatic nerve marking.

## SURGICAL TECHNIQUE

A detailed illustration of the surgical technique is shown in Video 1. Under general anesthesia, the patient is positioned prone on the flat operating table, and a pillow is placed under the lower abdomen to maintain the hip in slight flexion. The sciatic nerve is first marked preoperatively and is subsequently visualized using ultrasound (APLIO i700, Canon Medical Systems, Japan) at the level of the quadratus femoris muscle. A 23‐gauge needle is then inserted from the lateral aspect, and indigo carmine is injected around the sciatic nerve (Figure 1). For nerve marking, approximately 0.1 mL of indigo carmine diluted 10‐fold with hyaluronic acid is used. The 30° endoscope is used for visualization. The ultrasound probe covered in a sterile sleeve is prepared for use in the surgical field. Under ultrasound guidance, an 18‐gauge needle is inserted from the distal aspect to just above the ischial tuberosity, and approximately 10 mL of normal saline is injected to create an extra‐articular working space (Figure 2). A 1‐cm skin incision is made at the needle insertion site, and the endoscope is introduced through this portal into the created space. Next, the second portal is made from the medial side of the ischial tuberosity. The ultrasound probe is rotated 90°, and a 23‐gauge needle is inserted from the medial aspect and advanced toward the tip of the endoscope under ultrasound guidance. The needle tip is simultaneously visualized by the endoscope (Figure 3). A 1‐cm incision is made at this site, and a radiofrequency device is inserted through this portal. Because radiofrequency is located just above the ischial tuberosity, the ischial tuberosity can be reached with minimal soft‐tissue dissection. Similarly, a third portal is created from the lateral side of the ischial tuberosity by inserting another 23‐gauge needle toward the tip of the endoscope under ultrasound guidance (Figure 4). The sciatic nerve is identified by dissection toward a deeper layer along the lateral side of the ischial tuberosity. The prior indigo carmine marking of the sciatic nerve greatly facilitates its identification.

**FIGURE 1:**
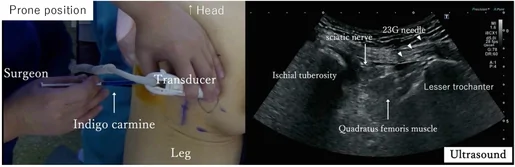
The sciatic nerve is visualized using ultrasound at the level of the quadratus femoris muscle. A 23‐gauge needle is inserted from the lateral aspect, and indigo carmine is injected around the sciatic nerve.

**FIGURE 2:**
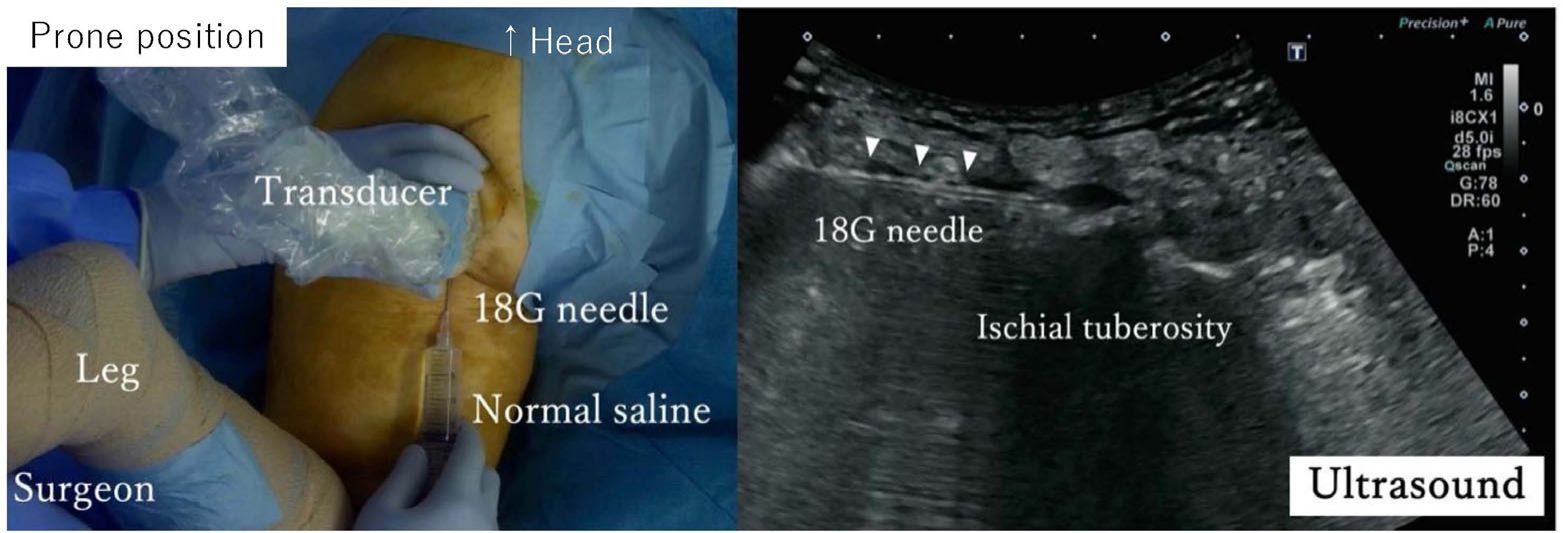
An 18‐gauge needle is inserted from the distal aspect to just above the ischial tuberosity, and normal saline is injected to create an extra‐articular working space.

**FIGURE 3:**
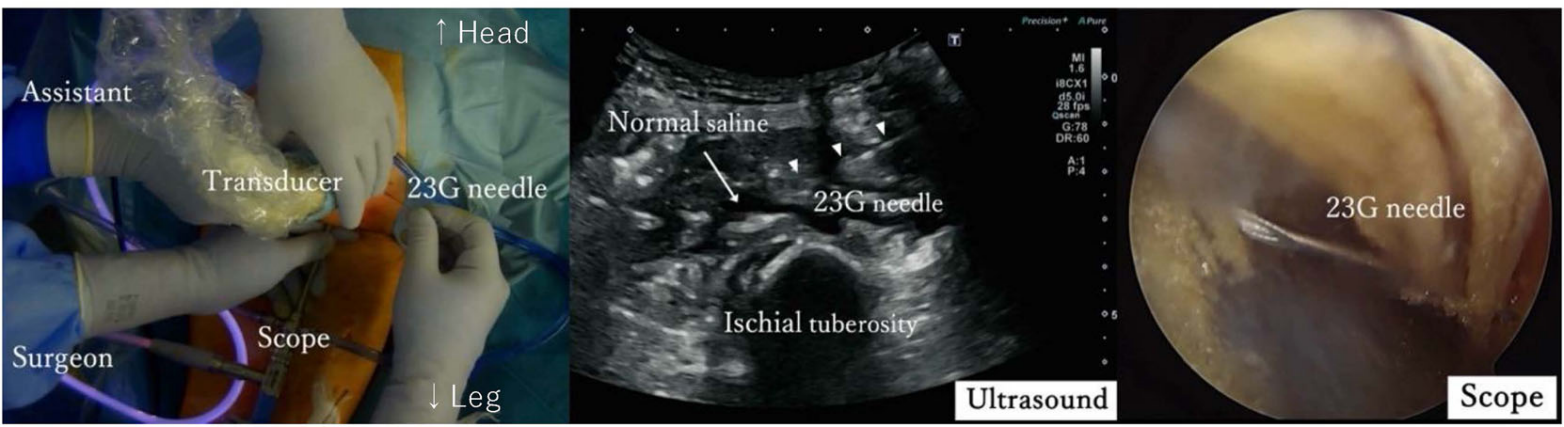
A 23‐gauge needle is inserted from the medial aspect, and the needle is advanced toward the tip of the endoscope under ultrasound guidance. The needle tip is visualized simultaneously with the endoscope.

**FIGURE 4:**
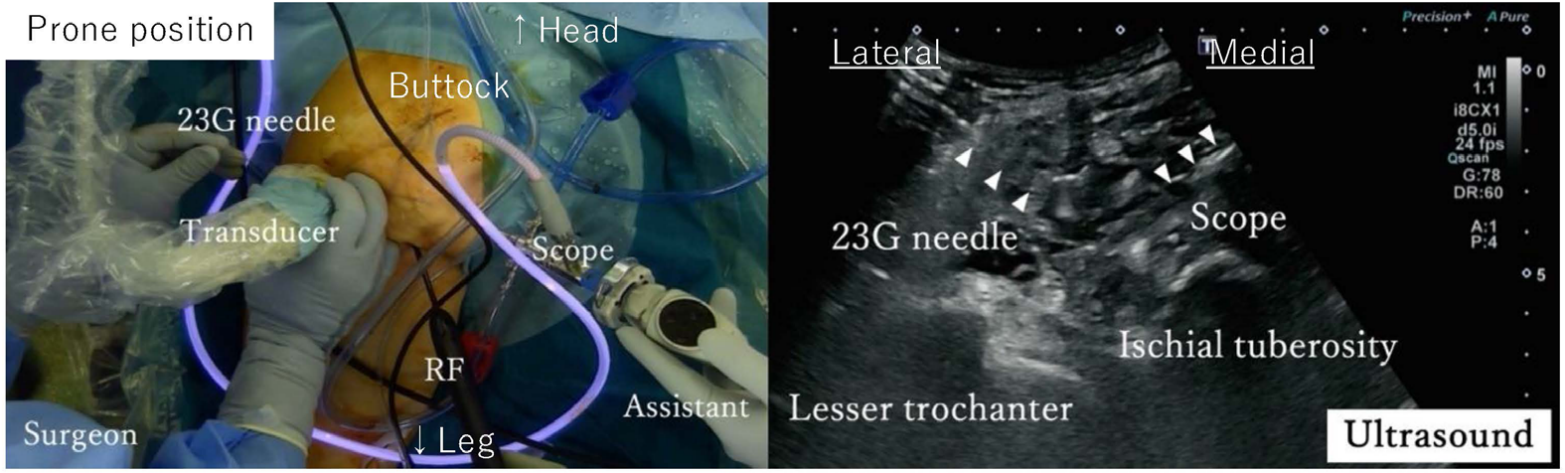
Another 23‐gauge needle is inserted from the lateral side of the ischial tuberosity toward the tip of the endoscope under ultrasound guidance. (RF, radiofrequency.)

A band of fibrous tissue is found superficial to the sciatic nerve and is compressing the nerve. Accordingly, the band is resected for decompression (Figure 5). The sciatic nerve is dissected and mobilized, and the underlying quadratus femoris muscle comes into view. A switching rod inserted through the viewing portal is used to retract the sciatic nerve medially, protecting it and creating a safer corridor for the remaining procedure. Using radiofrequency, the quadratus femoris muscle is released from its attachment on the lesser trochanter, which exposes the posterior surface of the lesser trochanter. The iliopsoas muscle attached to the anterior aspect of the lesser trochanter is also released (Figure 6). At this stage, the entire lesser trochanter is exposed, and an abrader (4.0 mm, Elite Acromioblaster, Smith & Nephew, Japan) is used to resect the lesser trochanter (Figure 7). Sufficient resection of the lesser trochanter is confirmed by fluoroscopy, and the procedure is completed.

**FIGURE 5:**
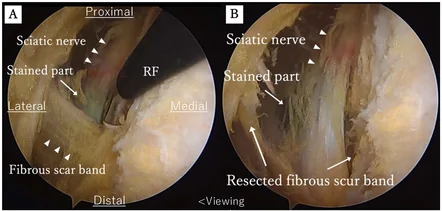
A band of fibrous tissue is found superficial to the stained sciatic nerve (A). This band is resected for decompression (B). (RF, radiofrequency.)

**FIGURE 6:**
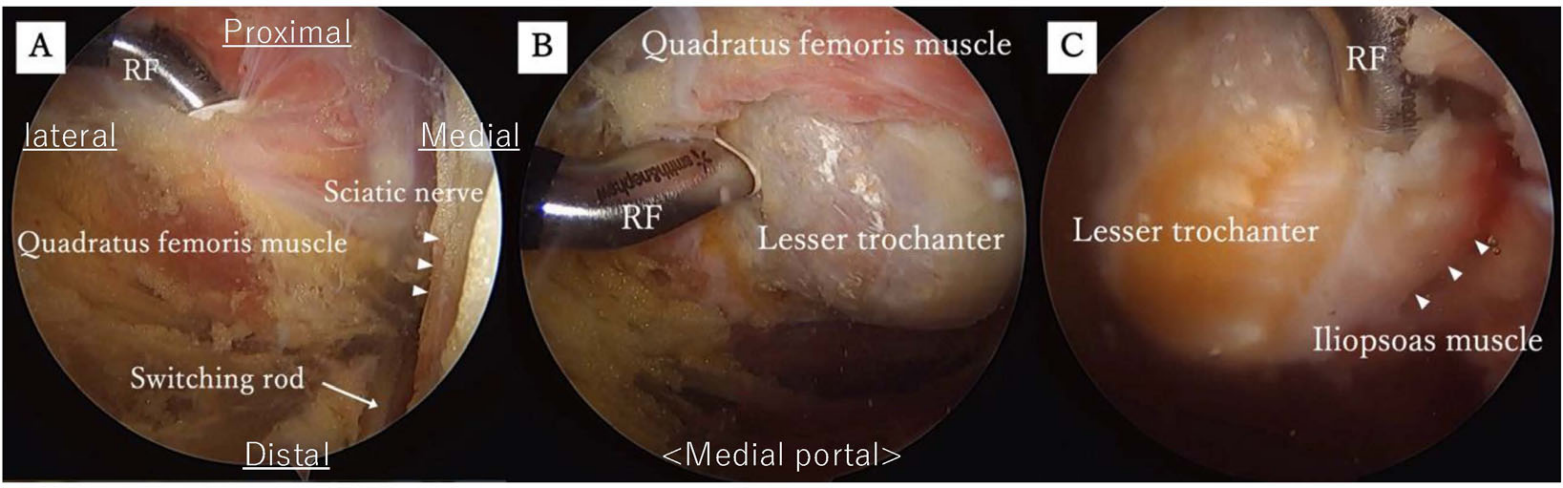
The sciatic nerve is dissected and retracted medially by a switching rod, and then the underlying quadratus femoris muscle comes into view (A). The quadratus femoris muscle is released from its attachment on the lesser trochanter, which exposes the posterior surface of the lesser trochanter (B). The iliopsoas muscle attached to the anterior aspect of the lesser trochanter is also released (C). (RF, radiofrequency.)

**FIGURE 7:**
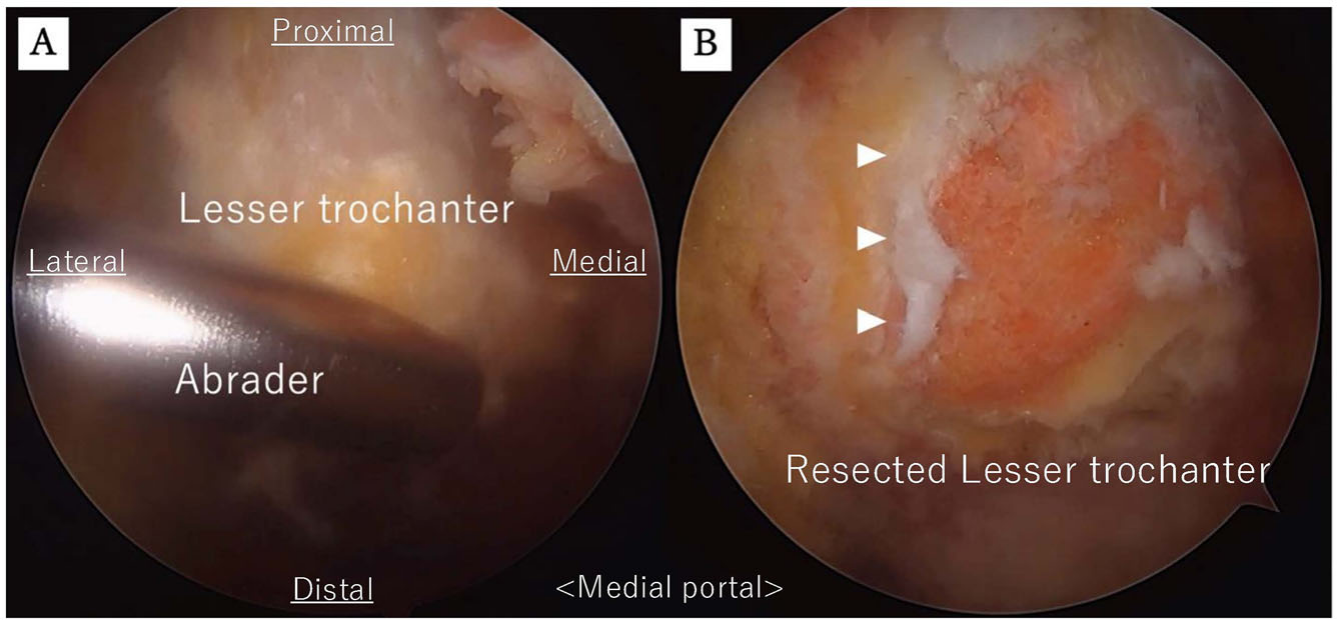
The entire lesser trochanter is exposed (A) and resected by abrader (B).

## DISCUSSION

IFI is an uncommon disorder, and reports of surgical treatment are limited. Endoscopic resection of the lesser trochanter to decompress IFS is regarded as an effective surgical treatment option.^3^ Hernandez et al. reported favorable clinical outcomes in a systematic review of endoscopic lesser trochanter resection for IFI.^4^

One of the major concerns associated with endoscopic lesser trochanter resection in the prone position is the risk of sciatic nerve injury. Therefore, accurate identification and protection of the sciatic nerve are the most important aspects of this procedure and can be achieved by safely creating optimal visualization. However, extra‐articular endoscopic surgery provides few distinct anatomic landmarks, and blind maneuvers carry a risk of neurovascular injury. Ultrasound‐assisted surgery, which can clearly delineate nerves intraoperatively, has emerged as a valuable adjunct to prevent such complications.^5^, ^6^ Ultrasound guidance allows the surgeon to simultaneously visualize surgical instruments and critical structures (nerves, vessels, and muscles) in real time, enabling the procedure to be performed more safely and with greater confidence. For endoscopic lesser trochanter resection, creating a portal under ultrasound guidance helps to safely secure a favorable endoscopic view.

Additionally, premarking the sciatic nerve with indigo carmine makes intraoperative nerve identification easier and more reliable. Moreover, it permits more extensive yet safe expansion of the working space as needed (Table 1). Care should be taken to avoid excessive use of indigo carmine because leakage into the surrounding tissues may obscure the operative field (Table 2).

**TABLE 1:** Advantages and Disadvantages

**Advantages**	**Disadvantages**
Endoscope allows for minimally invasive surgery	Both endoscope and ultrasound techniques require a learning curve
Ultrasound‐assisted technique enables accurate and safe portal placement	
Nerve marking facilitates easier identification and protection of the sciatic nerve	

**TABLE 2:** Pearls and Pitfalls

**Pearls**	**Pitfalls**
Perform nerve marking using a small volume of solution injected precisely around the nerve	Leakage of the dye used for nerve marking may obscure the operative field
Use ultrasound to continuously identify not only anatomical landmarks but also the position of surgical instruments	Accurate visualization with ultrasound may be difficult in obese patients

This ultrasound‐assisted technique does not require any specialized equipment and can be performed with standard ultrasound machines available in most institutions. Furthermore, the nerve marking method used here can be applied to any other nerve, indicating the high versatility of the procedure. Ultrasound assistance is an effective adjunct for enhancing the accuracy and safety of arthroscopic/endoscopic procedures, and wider adoption of these techniques can be expected in the future.

## DECLARATION OF GENERATIVE AI AND AI‐ASSISTED TECHNOLOGIES IN THE WRITING PROCESS

During the preparation of this work, the authors used Canva voice generation AI (version 4.195.1) to create narration for Video 1.

## DISCLOSURES

The authors (Y.M., K.M., Y.K., H.A., S.T., Y.I.) declare that they have no known competing financial interests or personal relationships that could have appeared to influence the work reported in this article.
